# Asymmetric Switching in a Homodimeric ABC Transporter: A Simulation Study

**DOI:** 10.1371/journal.pcbi.1000762

**Published:** 2010-04-29

**Authors:** Jussi Aittoniemi, Heidi de Wet, Frances M. Ashcroft, Mark S. P. Sansom

**Affiliations:** 1 Department of Biochemistry, University of Oxford, Oxford, United Kingdom; 2 Department of Physiology, Anatomy & Genetics, University of Oxford, Oxford, United Kingdom; National Cancer Institute, United States of America

## Abstract

ABC transporters are a large family of membrane proteins involved in a variety of cellular processes, including multidrug and tumor resistance and ion channel regulation. Advances in the structural and functional understanding of ABC transporters have revealed that hydrolysis at the two canonical nucleotide-binding sites (NBSs) is co-operative and non-simultaneous. A conserved core architecture of bacterial and eukaryotic ABC exporters has been established, as exemplified by the crystal structure of the homodimeric multidrug exporter Sav1866. Currently, it is unclear how sequential ATP hydrolysis arises in a symmetric homodimeric transporter, since it implies at least transient asymmetry at the NBSs. We show by molecular dynamics simulation that the initially symmetric structure of Sav1866 readily undergoes asymmetric transitions at its NBSs in a pre-hydrolytic nucleotide configuration. MgATP-binding residues and a network of charged residues at the dimer interface are shown to form a sequence of putative molecular switches that allow ATP hydrolysis only at one NBS. We extend our findings to eukaryotic ABC exporters which often consist of two non-identical half-transporters, frequently with degeneracy substitutions at one of their two NBSs. Interestingly, many residues involved in asymmetric conformational switching in Sav1866 are substituted in degenerate eukaryotic NBS. This finding strengthens recent suggestions that the interplay of a consensus and a degenerate NBS in eukaroytic ABC proteins pre-determines the sequence of hydrolysis at the two NBSs.

## Author Summary

ABC transporters are a large family of membrane proteins present in all organisms. Typically, they utilize ATP hydrolysis, the most prominent biological energy source, to translocate substrates into cells (e.g., bacterial nutritient uptake) or out of cells (e.g., multidrug exporters that contribute to antimicrobial resistance in bacteria and resistance to chemotherapeutic drugs in cancer). Also clinically relevant non-transport roles have been identified among ABC proteins. ABC transporters bind two molecules of ATP but do not hydrolyze them simultaneously. Therefore, an ABC transporter that consists of two symmetric halves must temporarily adopt asymmetric conformations at the two ATP-binding sites. Such transient conformational changes are difficult to address biochemically, but may be amenable to study by simulation methods, leading to future experiments. We employ molecular dynamics simulations to study how asymmetric switching might occur in the homodimeric bacterial ABC multidrug exporter Sav1866. The simulations suggest a mechanism of conformational switching that encompasses the ATP-binding sites and their interface towards the substrate-binding site. We extend our findings to show how asymmetric residue substitutions may render the switching process non-stochastic in mammalian Sav1866-like ABC exporters. This contributes to ongoing discussions about the role of two dissimilar ATP-binding sites in clinically relevant ABC proteins.

## Introduction

ATP-binding cassette (ABC) transporters are a large family of membrane proteins that use MgATP hydrolysis to drive the import or export of solutes to or from the cytoplasm. They undertake a number of physiological roles, for example bacterial nutrient uptake, bacterial drug resistance, tumor drug resistance, and peptide secretion [1]. Other ABC proteins act as nucleotide-gated Cl^−^ channels (CFTR) [2], [3] or ion channel regulators (SUR) [4], [5].

**Figure 1:**
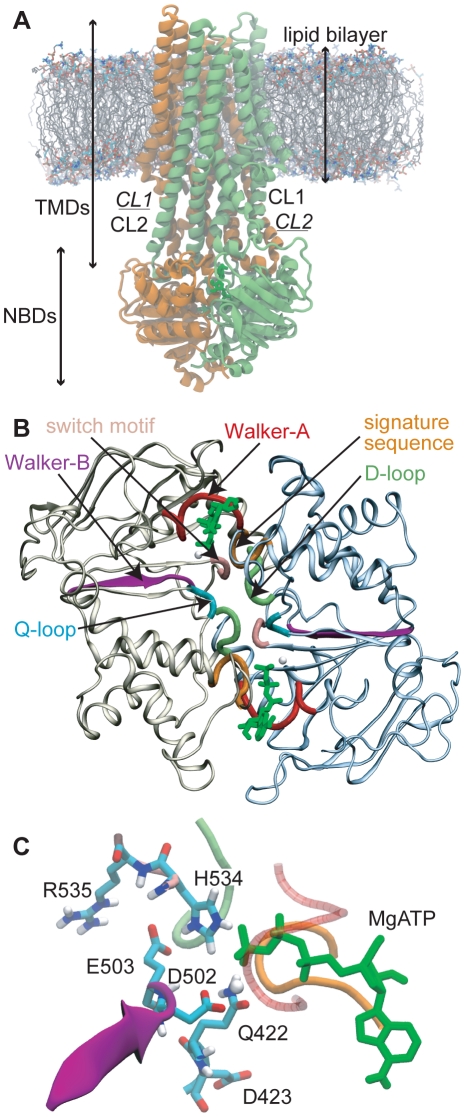
Structure of Sav1866. A: Sav1866 in a DPPC lipid bilayer. Each TMD contacts the NBDs by two cytosolic loops (CL1 and CL2, labeled in regular font for the green monomer and underlined italic font for the orange monomer). CL2 carries the coupling helix that is buried inside the NBD of the opposite monomer. B: Overview of the MgATP-bound NBD dimer, as seen from the TMDs. Functional motifs are indicated. ATP is colored green, Mg^2+^ brown. C: Close-up view of the MgATP binding site. Backbones of functional motifs are colored as in B. The signature sequence (orange) and the D-loop (lime) are provided by the other NBD. Selected residues are labeled.

Most ABC transporters consist of two transmembrane domains (TMDs) that provide a pathway across the membrane for the transported substrate, and two nucleotide-binding domains (NBDs) which form two nucleotide-binding sites (NBSs) at their dimer interface (see Figure 1) [6], [7]. In bacterial ABC importers the four constituent domains are distinct polypeptides. Bacterial exporters are typically formed by dimers of TMD-NBD half-transporters. Thus, most bacterial ABC transporters are formed of two identical TMDs and two identical NBDs, and feature consensus nucleotide binding/hydrolysis motifs at both NBSs. Such motifs are the Walker-A, Walker-B, Q-loop, signature and switch motifs, as illustrated in Figure 1B [8], [9].

ABC transporters are thought to hydrolyze the two MgATP molecules sequentially, as opposed to simultaneously, possibly involving a mechanism of alternating catalytic sites [9], [10]. This model implies that the NBD homodimer must adopt an *asymmetric* conformation when in a pre-hydrolytic nucleotide-bound configuration, at least transiently [11]. Such conformational switching to allow nucleotide hydrolysis at one or the other of the NBSs might be expected to occur stochastically. Indeed, asymmetric homodimers have been observed in crystal structures of the MgATP-bound NBDs of the ABC transporter HlyB [11]. Specifically, an intra-NBD salt bridge between a basic residue near the signature sequence (R611) and an acidic residue of the Q-loop (D551) was found to exist at only one of the two NBDs. An *inter*-NBD salt bridge involving the corresponding residues has also been shown to be functionally important in the eukaryotic ABC transporter Tap1/2 [12].

In contrast to homodimeric bacterial ABC proteins, many eukaryotic ABC transporters consist of two asymmetric halves, often resulting in one consensus and one degenerate NBS. The degenerate NBS typically displays markedly reduced ATPase activity compared to the consensus NBS [13]–[15]. Recent experiments suggest that such asymmetry can lead to the consensus NBS being first to hydrolyze ATP, followed by the degenerate site (e.g. in MDR3, Tap1/2) [16], [17]. Such a preferential order of ATP hydrolysis at the two NBSs has been proposed to help to control the transport of particularly complex substrates [16],[18]. Degenerate NBSs have been identified in several eukaryotic ABC transporters, including MDR3, TAP1/2, MRP1, CFTR, and SUR [14]–[17], [19].

In this work we use molecular dynamics (MD) simulations to explore the transient asymmetry that a homodimeric ABC exporter may adopt when in a pre-hydrolytic, MgATP-bound configuration. We simulate the bacterial multidrug exporter Sav1866, which is thought to represent the core architecture of ABC exporters [20], [6], [21], and has been used widely as a template for modeling of eukaryotic exporter structures [22], [23], [18], [24].

A number of other ABC transporters (e.g. BtuCD [25], [26]) and their NBDs (e.g. [27]–[32] have been subjects of MD simulation before, as such simulations can provide a valuable tool for exploring the conformational dynamics of channel and transport proteins in relation to function [33], [34]. It is now well established that MgATP binding/unbinding to the NBS closes/opens the NBD dimer, and that the helical subdomain of a NBD can rotate relative to its catalytic core domain [27], [28], [30], [32], [35]. However, it is unclear how a closed symmetric NBD dimer undergoes asymmetric transitions, and how these are affected by and linked to the TMDs.

Our multiple MD simulations show that an initially symmetrical MgATP-bound state of Sav1866 exhibits rapid (initial steps on ∼10 ns timescale) and stochastic switching into asymmetric NBD conformations. The NBDs seemingly adopt hydrolytically favorable conformations in only one NBS. We further show how the switching at the NBS is reflected in a network of charged residues at the TMD-NBD interface. We extend our observations of stochastic switching in symmetric NBDs to suggest how degeneracy in eukaryotic ABC exporters may lead to preferential switching. This provides a rationale for understanding residue substitutions in ABC proteins, such as CFTR, MRP1, Tap1/2 and SUR.

## Methods

### System Preparation

Polar hydrogens were added to the ADP-bound crystal structure of Sav1866 (pdb id 2HYD) using GROMACS [36], [37]. A doubly protonated state was chosen for the side chain of H534. Default protonation states were assumed for all other residues. MgATP was modeled on the bound ADP by positioning the γ-phosphate in line with the α- and β-phosphate moieties followed by careful energy minimization. The Mg^2+^ ion was positioned next to the β- and γ-phosphates towards the Walker-B motif. In the default ATP force field the γ-phosphate is singly protonated. We modified this by removing the hydrogen from the γ-phosphate and redistributing the partial charges evenly over the phosphate oxygen atoms such that the overall charge of the MgATP is -2e.

The protein was positioned manually in a preformed DPPC bilayer containing 512 lipid molecules. A shell of lipid molecules around the protein was removed, and the remaining 376 lipids were relaxed around the protein by a 0.5 ns simulation with position restraints on all non-hydrogen protein and MgATP atoms (harmonic restraints, force constant 1000 kJ mol^−1^ nm^−2^). In a similar fashion, we inserted the Sav1866 structure into a POPC bilayer. A snapshot of the bilayer-inserted Sav1866 structure is shown in Figure 1A. The position restrained simulations should allow for water to enter the nucleotide-binding sites. The Sav1866 crystal structure itself contains no waters near the ADP phosphate moieties.

### MD Simulations and Analysis

MD simulations were performed using the GROMACS 3.3.1 software [36], [37] and the ff53a6 version of the GROMOS96 force field [38], [39]. The simulation box adopted dimensions of 118×118×185 Å, and was solvated by 60750 water molecules, using the single point charge (SPC) water model [40]. The system was energy minimized by 1000 steps of steepest descent. After the position-restrained run (see above), we performed a warm-up simulation across a temperature interval of 100 K–310 K in steps of 50 K and 50 ps simulation. Production runs were performed with a time step of 2 fs. The particle mesh Ewald summation method [41] with a real space cut-off of 12 Å was used to calculate long-range electrostatic interactions. Hydrogen bonds were analyzed using a geometric definition, namely a maximum donor-acceptor distance of 3.5 Å, and a maximum acceptor-donor-hydrogen angle of 30°. Atomic contacts between two residues were defined individually for each simulation snapshot as the number of atom pairs between the two residues whose positions are closer than 4 Å. Images were prepared using the programs VMD [42], ClustalX [43], xmgrace, and MATLAB (The MathWorks, Inc.). We also included snapshots of the simulations as a PDB file (see supplementary Text S1) containing the protein, MgATP, and 40 water molecules that are closest to the MgATP molecules. The snapshots are from one simulation at 0, 5, 10, 20, 30 and 40 ns.

We first performed six repeat simulations of Sav1866 with MgATP nucleotide in a DPPC lipid bilayer, each with different starting velocities and a duration of 40 ns. In order to probe the effects of the lipid environment in our simulations, we also performed a 30 ns simulation of Sav1866 in a POPC bilayer. In addition, we performed two further control simulations with bound ADP and DPPC lipid, both to a duration of 30 ns.

## Results

### Overall Simulation Quality and Effect of Lipid Environment

**Figure 2:**
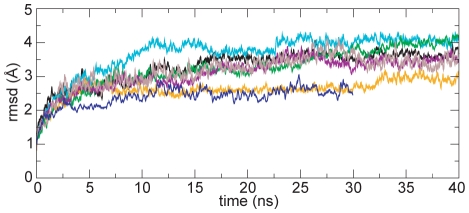
Structural stability. Root-mean-square deviations of C-alpha atoms in six repeat simulations with DPPC bilayer (40 ns duration), and one repeat with POPC bilayer (30 ns duration).

Root-mean-square deviations (rmsd) of Cα atoms in our six simulations with DPPC lipid and one simulation with POPC lipid are within typical values observed for protein simulations: 2.5–4 Å for the whole protein, and 2–2.5 Å for individual NBD subunits (see Table 1). Given the relatively large size of dimeric Sav1866 (1156 aa), rmsd values in this range are indicative of structural stability. The variation seen between graphs of rmsd vs. time of individual repeats (Figure 2) might be indicative of distinct, stochastic conformational changes. This would suggest that different repeat simulations may cover different regions of the accessible conformational space. However it should be kept in mind that the variation in rmsds is within experimental limits for a protein of this size.

**Table 1:** Root mean square deviations (rmsd, in Å) of C-alpha atoms of different domains of the Sav1866 dimer relative to the beginning of the simulation.

simulation	whole protein	NBD A+B	NBD A	NBD B	TMD A+B	TMD A	TMD B
DPPC 1	3.6	2.5	2.2	2.5	3.9	3.2	4.3
DPPC 2	2.9	2.3	2.1	2.2	2.6	2.3	2.5
DPPC 3	4.1	2.7	2.4	2.6	4.1	3.8	4.4
DPPC 4	3.9	2.1	2.0	1.9	4.4	4.3	4.5
DPPC 5	3.5	2.4	2.1	2.4	3.4	3.4	3.0
DPPC 6	3.4	2.6	2.4	2.3	3.1	2.6	3.2
POPC	2.6	2.1	2.0	1.9	2.1	2.2	2.1

The domain-specific rmsd values for the NBDs in the 30 ns simulation performed with POPC lipid are within the range observed in the 40 ns simulations with DPPC lipid, but those of the TMDs are lower in the POPC simulation (Table 1). This indicates that the different lipid environments may have an effect on the TMD conformation. However, the rmsd of the whole protein vs. time for the POPC simulation falls within the range defined by the other simulations. Further, considering the high overall stability of the protein and the identical rmsd values seen for the NBDs, it seems very unlikely that the lipid environment would have any effect on the cytosolic regions of Sav1866 during the time covered in our simulations. Therefore, we chose to treat the POPC simulation as a seventh repeat, and collated data derived from it with that from the six DPPC simulations.

### Distinct MgATP Binding Modes

**Figure 3:**
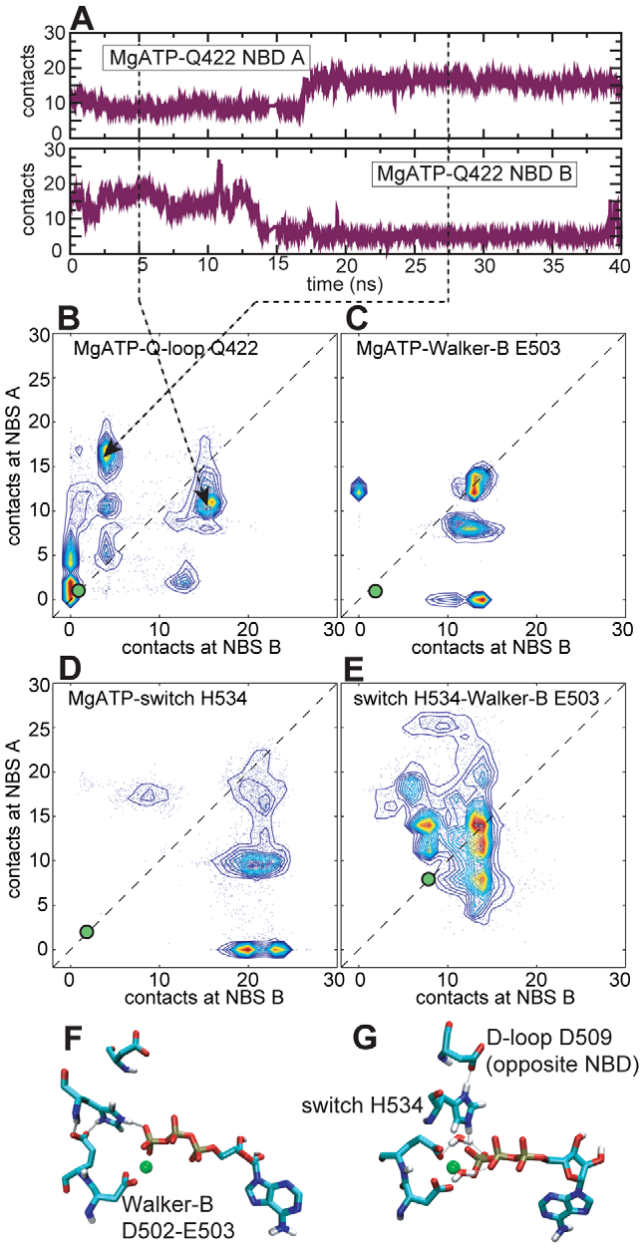
Interactions at the MgATP binding site. B–E: Inter-residue contact numbers (4 Å cut-off) of selected residues at the MgATP binding sites visualized in scatter plots with the two NBS on the two axes. Higher numbers of contact are indicative of stronger interactions. Scatter plots were generated from simulation snapshots in 50 ps intervals (see SI Text S2 for more detail). Contour lines indicate frequently populated configurations (red: most frequent, blue: less frequent). The contacts observed in a protonated form of the ADP-bound Sav1866 crystal structure are indicated by green dots. A: MgATP-Q422 contacts shown in a time-resolved plot for one of the six simulations. F, G: Simulation snapshots of MgATP binding.

To identify possible binding modes for MgATP at the two NBSs we determined atomic contacts between MgATP and the most important nucleotide-binding residues. In addition to inspecting various contacts as a function of time for each simulation (e.g. Figure 3A, SI Figure S1), we analyzed contacts over an ensemble of seven simulations. In particular, we compared patterns of atomic contacts (using a 4 Å cut-off) at the two NBSs of the protein via scatter plots with contact numbers at the two NBS on the two axes (Figure 3B–E). Such a representation allows for visualization of possible asymmetry between the two NBSs, seen as off-diagonal elements in the scatter plot. It should be noted that, in our definition, closely interacting residues will yield more than one atomic contact. Therefore, the number of contacts is not only an indication of the existence of an interaction, but also a measure of its strength.

From this analysis, MgATP appears to form several distinct binding modes (Figure 3). For example, MgATP and the Q-loop Gln residue (Q422) show three levels of interaction, exhibiting either ∼5, 10–15, or >15 contacts (Figure 3B). These levels arise from distinct interaction patterns, corresponding to coordination of the Mg^2+^ ion by the Gln oxygen atom, H-bonding of the Gln amide to ATP phosphate oxygen atoms, or both interactions together. An orientation of the Q-loop Gln that encompassed this range of Gln-MgATP interactions was observed in the MgAMP-PNP-bound crystal structure of the Rad50 ATPase domain, which is homologous to ABC transporter NBDs [44]. The Q-loop-MgATP association in our simulations supports the idea that the Q-loop Gln senses the presence of MgATP [45]. In comparison, Q422 forms no contacts to nucleotide in the ADP-bound crystal structure.

Distinct levels of contact are also seen between MgATP and the Walker-B Glu (E503; Figure 3C), resulting from involvement of E503 in Mg^2+^ coordination. The possibility of direct E503-MgATP interactions seen in our simulations adds to the conformational variability available to this crucial catalytic residue (see also below). It should be noted that we also observe direct interactions of the Walker-B aspartate D502 with Mg^2+^ (data not shown).

Our simulations also feature contacts of the switch motif H534 to MgATP (Figure 3D), arising through H-bonding of its ε-NH to the ATP γ-phosphate and electrostatic attractions. The switch motif His (H534) and the Walker-B motif Glu (E503) are known to form a functionally important “catalytic dyad” [46]. As can be seen (Figure 3E) this dyad forms stably in all simulations of the pre-hydrolytic state, and associates more tightly than in the (ADP-bound) crystal structure. The relatively wide distribution of contacts suggests a degree of conformational flexibility with low energetic barriers for the catalytic dyad (see also Figures 3F,G). In accordance with this idea, the catalytic dyad adopts various different conformations in existing ABC NBD crystal structures. We also find that the switch motif H534 can H-bond directly to the D509 side chain of the D-loop (SALD motif) of the opposite monomer (see Figure 3G). This is particularly interesting since the D-loop has been implicated in inter-NBD cross-talk in other ABC proteins [27], [47].

As noted above, the off-diagonal elements in the scatter plots of contact numbers (Figure 3B–E) indicate that asymmetric interactions are present in *all* examined residue and nucleotide pairs. That is, in individual simulations (or segments of simulations) one NBS can form more contacts than the other, indicative of stochastic switching in symmetric NBDs. However, the off-diagonal elements are not always equally distributed either side of the diagonal. This is to be expected of intrinsically stochastic switching, combined with incomplete sampling of the space of possible interactions in our simulations.

The ADP-bound simulations show hardly any interactions of the studied residues with the nucleotide (see SI Figure S7). This is not surprising, since the studied residues are mostly thought to interact with the gamma-phosphate moiety of ATP.

### Role of Q-loop in NBD-TMD Coupling

The conserved Q-loop motif has been implicated in distinguishing between ATP and ADP at the NBS [45]. Because of its location at the NBD-TMD interface, the Q-loop is also well positioned to relay conformational changes to the TMDs [20]. In Sav1866, D423 (of the Q-loop) and K483 (adjacent to the LSGGQ signature sequence) form inter-NBD charge pairs in the ADP-bound crystal structure. Likewise, the crystal structures of HlyB revealed a conserved aspartate of the Q-loop to form a charge pair with a basic residue next to the signature sequence. This intra-NBD charge pair was seen only at one NBS in MgATP-bound HlyB dimers [11]. Mutagenesis studies of the ABC transporter Tap1/2 suggested that the equivalent charge pair forms in an inter-NBD manner [12].

**Figure 4:**
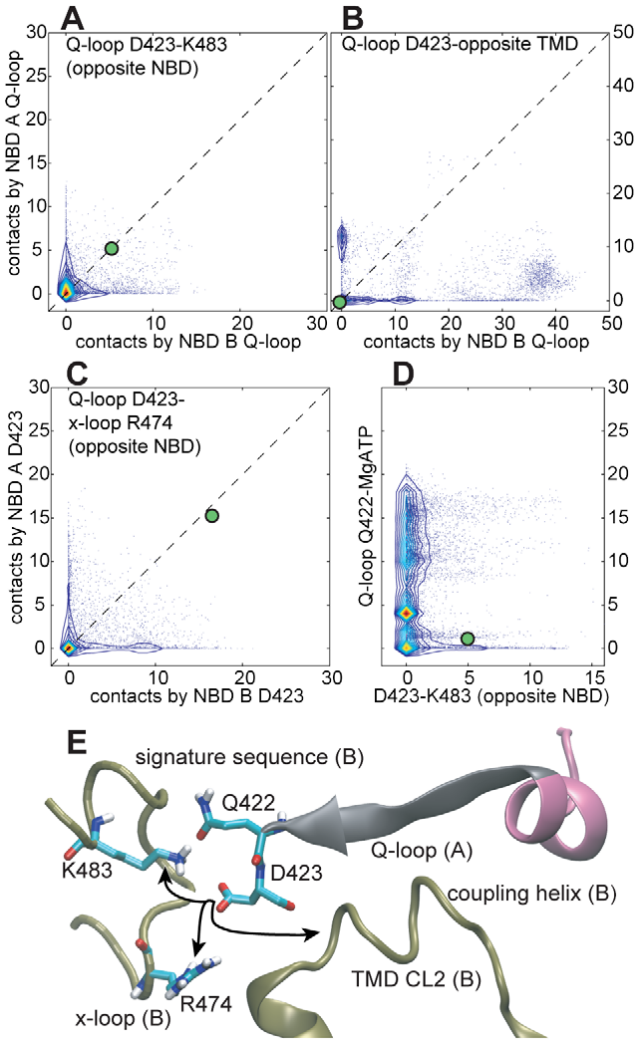
Interactions at the NBD-TMD interface. A–D: Scatter plots of contacts formed by the Q-loop D423. Contacts observed in the protonated Sav1866 crystal structure are indicted by green dots. Scatter plots were generated as described for Figure 3 and detailed in SI Text S2. Subfigure D combines data from both NBDs. E: Different interactions formed by D423. Regions contributed by different monomers are distinguished by color and labels: A (grey, pink), B (brown).

In our simulations, inter-NBD D423-K483 interactions were frequent (Figure 4A) whereas only very few intra-NBD charge pairs formed (data not shown). We also observed contacts between D423 and the TMDs (Figure 4B). It is evident from Figure 4A that the D423-K483 interaction does not occur simultaneously at both NBDs. Rather, there appears to be competition between K483 and the TMDs for interaction with D423. Thus configurations are frequent in which D423 of one monomer pairs with K483 of the other monomer, and D423 of the other monomer interacts with the TMDs (see time-resolved plots in SI Figure S2, and also SI Figure S6). In effect, dissociation from K483 frees D423 to interact with the TMDs. Furthermore, a scatter plot of the D423-K483 contacts versus the Q-loop Gln (Q422) contacts to MgATP indicates that a strong D423-K483 interaction coincides with the neighboring Q422 being either in a high-contact binding mode to MgATP, or being completely dissociated from MgATP (Figure 4D). This suggests that the conformation of the ATP-sensing Q422 may influence the interactions of its neighboring D423 with K483, and vice versa.

In the Sav1866 crystal structures, the D423 residues of each of the two Q-loops interact with the R474 residues of the so-called x-loops of the opposite NBDs (Figure 4C, see also below). These charge pairs dissociate in almost all of our simulations (Figure 4C, SI Figure S6) and D423 alternates between interacting with K483, R474 and the TMDs (Figure 4E, SI Figure S6). On most occasions, the D423 residues of the two monomers interact *asymmetrically* with these different residues.

Interactions formed by the Q-loop D423 in the ADP-bound simulations also show remarkable drift away from their contact numbers in the starting crystal structure, as shown in a scatter plot similar to that in Figure 4 (see SI Figure S8). Some of the D423 interactions seem to occur more readily at both monomers in the ADP-simulations than the MgATP-simulations.

### Asymmetric Switching at the NBD-TMD Interface

Sav1866 contains a short sequence motif (the x-loop) at the NBD-TMD interface that is specific to ABC exporters [20]. It has been suggested that the GERG motif of the Sav1866 x-loop connects the TMD cytosolic loops (CLs) in a nucleotide-dependent manner. To study the role of the GERG motif, we analyzed contacts of E473 and R474 to the four CLs in a similar fashion to the analyses presented above (see SI Figure S3). This analysis confirms the proposed link between the GERG motif and the CLs: R474 can bind to the CL2 of both TMDs, whilst E473 binds both to the CL1 of the TMD on the opposite monomer and, frequently, to CL2 of the TMD of its own monomer. Most of these interactions form asymmetrically, indicative of stochastic switching, but as already seen for the MgATP binding modes the conformational space is not completely sampled. Instead, the GERG motif of NBD B forms tight interactions to the TMDs more often than that of NBD A.

**Figure 5:**
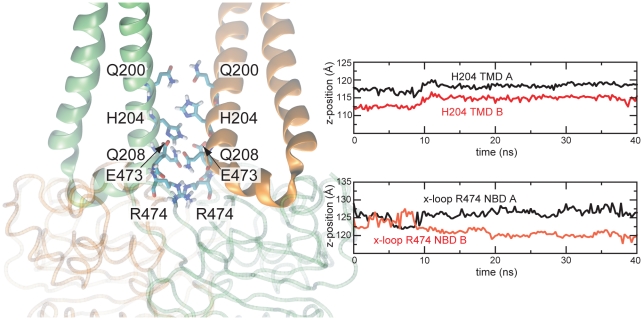
Initial asymmetry in the Sav1866 crystal structure. Residue pairs along the Sav1866 symmetry axis, at the NBD-TMD interface, in a protonated form of the crystal structure. The CL2 loops of the TMDs are shown as green and orange ribbons. The NBDs are shown as transparent C-alpha traces. Exemplary plots of z-coordinates (along the bilayer normal) of the x-loop R474 and H204 in one of the repeat simulations indicate asymmetric stacking. See SI Figure S4 for similar plots in all simulations.

**Figure 6:**
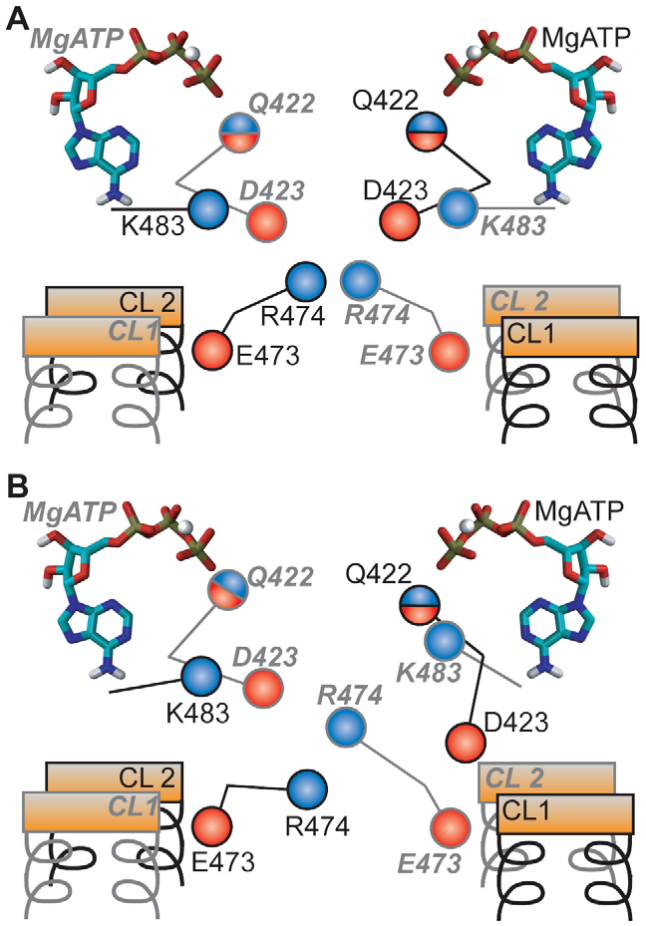
Electrostatic interactions at the NBD-TMD interface. A: Initial symmetric arrangement before simulation. B: Asymmetric arrangement observed in simulation, involving stacking of the two x-loop R474 and interactions of Q-loop D423 with the TMDs. Labels referring to the two monomers are distinguished by regular black font and bold-italic gray font.

The asymmetric GERG motif interactions with the NBDs arise from a stacking of the R474 pair on the Sav1866 symmetry axis. As indicated by the trajectory of the projection of R474 onto the lipid bilayer normal from one simulation (Figure 5, see also SI Figure S4), the arginine residues start next to each other but in the simulations repack to stack on top of one another, breaking the symmetry (see also Figure 6). As a consequence, the GERG motif whose R474 sits closer to the TMDs forms more overall interactions with the TMDs (see also SI Figure S5).

The cytosolic ends of the TMDs, which form the interface to the x-loops, contain a series of residues along the dimer symmetry axis: Q208, H204, Q200 (see Figure 5). These pairs of residues start the simulations in asymmetric patterns: the pairs of glutamine residues face each other with the amine nitrogen atoms next to the oxygen atoms, while the pair of H204 is stacked along the symmetry axis. As indicated by time-resolved plots (Figure 5, SI Figure S4), the pairs of H204 remain stacked throughout the simulations.

## Discussion

### Mechanistic Implications

The simulations described above reveal a pronounced asymmetry between the dynamic behavior of two initially identical Sav1866 monomers, such that several key interactions are found to exist only at one monomer at a time. These interactions include a salt bridge (D423-K483) that has also been reported to form in only one monomer in crystal structures of the homologous MgATP-bound NBD homodimer HlyB [11]. Interestingly, while the HlyB study reported this salt bridge to be formed by residues *within* the same NBD monomer, it forms *between* the two NBDs in Sav1866. Similar *inter*-subunit charge pairing has also been reported for the Tap1/2 transporter [12]. The Tap1/2 study further suggested that the inter-NBD charge pairs are structurally coupled and thus tied to conformational changes anticipated during ATP hydrolysis [12].

Our simulations support this idea of a structural coupling of the inter-NBD Q-loop D423-K483 charge pair. This pairing may be dependent on the association of the Q-loop Q422 with MgATP. Furthermore, when D423 is unbound from K483, it can flip towards the TMDs and interact with the coupling helix on CL2 of the opposite monomer (Figure 4B). Interaction of D423 with CL2 can induce further tightening of NBD-TMD interactions, partly through clamping of the coupling helix by the β-strand on which D423 is located (Figure 4E). The formation of D423-K483 and D423-TMD interactions is preceded by breaking of the D423 charge pairs with R474 of the x-loop.

Another region in which we observe asymmetric switching is the GERG motif of the x-loop. Our simulations support its proposed role in linking the CLs together [20]. However, in simulations the GERG motifs of the two monomers can do so asymmetrically. This is a consequence of the pair of R474 rearranging along the Sav1866 central axis, with one R474 interacting tighter with the TMDs than the other. The complex interplay of charged and polar residues at the NBDs and across the NBD-TMD interface is summarized in Figure 6.

Interestingly, Dawson and Locher [48] reported small conformational differences in the x-loop between the ADP-bound crystal form of Sav1866 and a structure crystallized with the non-hydrolyzable ATP-analog AMP-PNP. However, they were uncertain about the significance of these differences because of the limited resolution of the AMP-PNP crystal structure [48]. Our results indicate that the x-loop rearranges readily between the ADP-bound and MgATP-bound forms. However, while the x-loops were modeled symmetrically into the ADP- and AMP-PNP-bound electron densities, our simulations indicate the possibility of an asymmetric arrangement along the dimer axis. The x-loop and K483 lie very close (in the primary sequence) to the LSGGQ signature sequence, which is important in MgATP binding and hydrolysis in ABC transporters. Therefore, asymmetric behavior at these regions may be linked to non-simultaneous hydrolysis activity at the two NBS.

Our observation of asymmetric switching in Sav1866 is in line with the asymmetric function of its NBDs. As a comparison to this finding, we sought for a protein that requires perfect symmetry for proper function. Selectivity filters of potassium channels are likely to be dependent on their tetrameric symmetry to ensure K^+^ conduction and selectivity. Using a simulation trajectory generated in our laboratory for another study, we examined an inter-residue interaction at a crucial position near the selectivity filter: that between (protonated) E71 and D80. The E71-D80 interaction has been shown to be involved in slow inactivation of KcsA, underlining its functional importance [49]. We found this interaction to be near constant at all four monomers (SI Figure S9). Albeit anecdotal, this finding provides a control example in which functional requirements seem to result in preservation of symmetry during the course of a simulation.

### Asymmetry in the TMDs and Possible Switching Role of Substrate Binding

In the structure determination of ADP-bound Sav1866, two-fold non-crystallographic symmetry was applied to large parts of the dimer, although some pairs of residues were refined in non-symmetric conformations [20]. Therefore, the starting structure in our simulations was not perfectly symmetric.

While most asymmetric residue pairs are located on the outside of the Sav1866 dimer and presumably contribute to crystal packing, a cluster of initially asymmetric residues is found at the cytosolic end of the TMDs, at the dimer interface along the symmetry axis (see Figure 5). This cluster includes the two H204 residues, which are positioned on the symmetry axis one above the other (SI Figures S4, S5). As a consequence of their stacking rearrangement, one of the two R474 residues can interact with H204 of the opposite monomer. Therefore, the starting asymmetry at H204 (H204 of TMD A closer to the NBDs) might have biased the simulations towards cases in which R474 of NBD B ends up closer to the TMDs and interacts with H204 of TMD A. The asymmetric arrangement of H204, as well as Q200 and Q208, along the symmetry axis may effectively constitute another molecular switch in Sav1866.

It should also be noted that our simulations were performed in the absence of a transported substrate. The binding of substrate is known to promote ATPase activity in ABC transporters including Sav1866 [21]. It is possible that, in the physiological transport cycle, substrate binding to Sav1866 induces conformational changes at the x-loop that render MgATP binding and hydrolysis more favorable at the NBDs. The homodimeric Sav1866 presents two symmetric binding sites to substrates. Therefore, the conformational changes induced by the substrate may also carry information (possibly via Q200-H204-Q208 and the x-loop) about which NBS should hydrolyze first in order to optimize translocation of the substrate in its current binding mode.

### Choreography of an Asymmetric Switching Process

Our simulations were set up by changing the nucleotides in the pseudo-symmetric post-hydrolytic Sav1866 structure into a model of the pre-hydrolytic nucleotide configuration. This allowed us to study possible first steps of a putative pre-hydrolytic switching. We observe asymmetric structural rearrangements relative to the ADP-bound crystal structure, suggesting that the substitution of MgATP into the binding sites initiated a switching process. However, since our simulations are relatively short (and do not allow for formation or breaking of covalent bonds), we cannot expect to see beyond the initial steps of this process.

We observe several regions of asymmetric switching. Firstly, the MgATP-sensing Q-loop Q422 forms a high-contact MgATP binding mode typically only at one NBS. Secondly, the neighboring Q-loop residue D423 charge pairs with a basic residue (K483) near the signature sequence only at one NBS at a time, as has been reported in the ABC transporters HlyB and Tap1/2 [11], [12]. D423 that is not bound to K483 is free to interact with the TMD coupling helices and with R474 of the x-loop GERG motif. Thirdly, the x-loop GERG motif can undergo asymmetric stacking along the Sav1866 symmetry axis, such that R474 of one monomer interacts much more tightly with the TMDs than R474 of the other monomer. Finally, the catalytic dyad E503-H534 frequently associates in different ways at the two NBSs. A schematic diagram of these asymmetric interactions in the simulations is shown in supplementary Figure S6.

Conceivably, the asymmetric interactions in the Sav1866 NBDs effectively constitute a series of molecular switches (Figures 6, S6). To complete the asymmetric switching that leads to hydrolysis at one of the NBSs, each of these switches may have to adopt a certain state. Such a series of switches may thereby create a complex, multi-state energy landscape for the conformational transition in pre-hydrolytic Sav1866. It should be remembered that our simulations sample only about 270 ns of Sav1866 dynamics in this energy landscape, while full transport events probably occur on time scales of ≥10^−3^ s. Therefore it would be statistically unlikely to observe large conformational transitions of Sav1866. Even the probable first step of such transitions may be too unlikely to observe, ie, all of the molecular switches to adopt a state needed to complete the initial asymmetric switching.

Ultimately, the behaviour of Sav1866 appears stochastic at the level of these molecular switches. A picture emerges in which, at the atomic level, ABC transporters do not function as molecular motors with perfectly defined steps. Rather, coincidental stochastic steps underpin the larger-scale motions required for function. Therefore, our sequence of simulation snapshots (SI Text S1) should not be interpreted as a strictly temporal sequence of conformational change (see also Figure S6). It should be noted, though, that this picture may be subject to change in the presence of transported substrate, which is missing in our simulations.

### Methodological Considerations

Many interactions in our system showed considerable variability in contact numbers and possible binding modes between individual runs within the ensemble of seven simulations. It seems likely that substitution of ADP for MgATP at both NBSs left the initial system in an energetically activated (i.e. non-equilibrium) state, thus enhancing the extent of conformational space accessible in relatively short (40 ns) simulations. Thus, our approach of repeated MD simulations helps to extend the range of conformations accessed. However, our simulations provide far from exhaustive coverage of the conformational space. This is apparent in the lack of diagonal symmetry in many of the scatter plots of contact numbers. Asymmetric coverage of conformational space could also be indicative of an inherent bias in the starting structure, such as the asymmetric starting orientations of residues along the symmetry axis (see above).

In NBD crystal structures with Mg-nucleotides, interactions of the Walker-B aspartate with Mg^2+^ are water-mediated. This suggests that our protocol of solvating the MgATP may have been insufficient (see Methods), and that our simulations may be missing a water molecule between the Walker-B motif and the Mg. Effectively, our hydration protocol may have resulted in an interchange of parts of the first and second hydration shells of Mg^2+^ (namely, the Walker-B aspartate and water). Otherwise, our simulations feature quick rearrangements of the NBD residues to accommodate for the MgATP, for example in case of the “ATP-sensing” Q-loop Q422. However, it should be noted that it remains beyond this study to establish a single physiologically correct MgATP binding mode in Sav1866 and ABC exporters in general. Binding modes of ATP and ATP analogs vary between crystal structures of ABC NBDs (e.g. Tap1 NBD [17] and FbpC [35]) and may thus be expected to vary in simulations as well.

Finally, we wish to note that our method of visualizing atomic interactions in scatter plots provides a compact and visually intuitive picture of the distinct interaction patterns and the arising asymmetry accessed by our repeat simulations. Similar methods of visualization may be of help in future MD simulation studies, especially as it looks to become more common to extend phase space coverage by repeat simulations.

### Implications for Eukaryotic ABC Transporters

**Figure 7:**
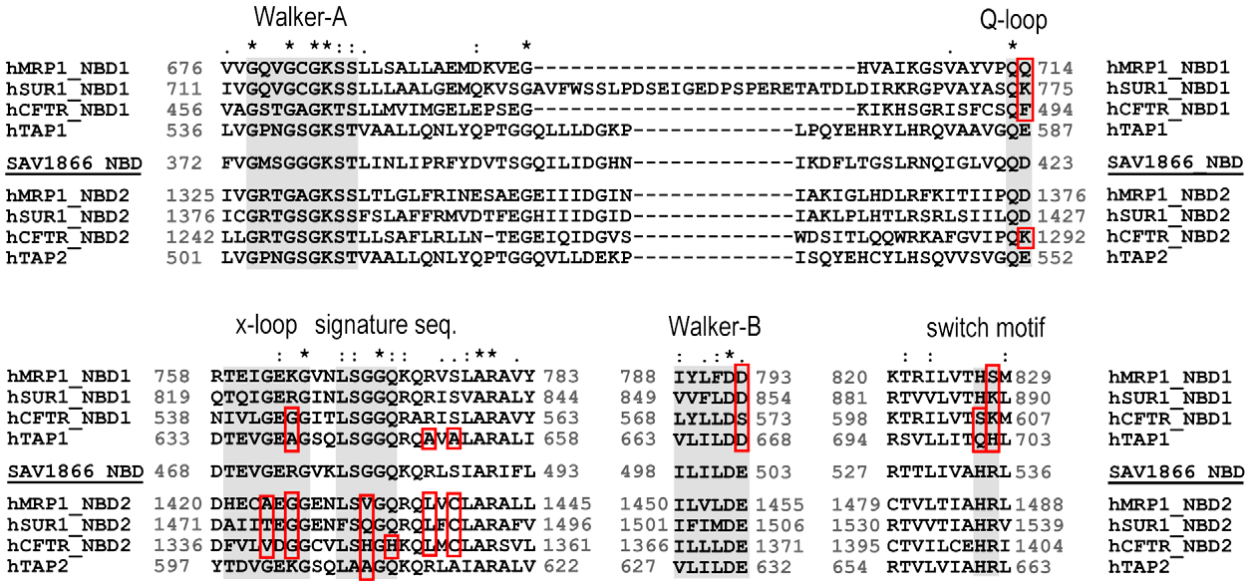
Degeneracy in eukaryotic ABC proteins. Sequence alignments of conserved NBD motifs in Sav1866 and selected eukaryotic ABC proteins. Asterisks indicate identical residues. Colons/dots indicate closely/less similar residues. Degeneracy substitutions in functional motifs are highlighted by surrounding boxes.

Although our simulations have been performed on a homodimeric bacterial ABC transporter, the results have possible implications for its (more complex) eukaryotic counterparts. Eukaryotic ABC transporters are often encoded with the two half-transporters in one protein, thus allowing for sequence and (potential) functional differences between the two halves. Indeed, eukaryotic ABC proteins often contain one “consensus” NBS and one “degenerate” NBS [17] with substitutions at the functional motifs (Figure 7). This holds in particular for the ABCC subfamily [19], [17], [12]. Strikingly, residues that seem to act as molecular switches in our simulations are frequently degenerate. Thus, the Q-loop Asp (D423 in Sav1866) is degenerate in NBD1 of the ABCC-type proteins MRP1, SUR1, and CFTR. The arginine of the x-loop GERG motif (R474 in Sav1866) is degenerate in NBD2 of all ABCC transporters, the Tap1 half of the Tap1/2 transporter, and NBD1 of CFTR. Because of the domain-swapped NBD interface, the substitution of the x-loop in NBD2 of ABCC transporters adds to their degeneracy at NBS1. The Walker-B glutamate (E503 in Sav1866), which is intimately involved in the intricate catalysis chemistry, is degenerate at NBD1 of the ABCC transporters. Further degeneracy substitutions are found at the signature sequence, and the switch motif of NBD1 (Figure 7).

It has been suggested before that the combination of one consensus and one degenerate NBS may lead to *directed switching* in the pre-hydrolytic conformation [16], [17]. Eukaryotic ABC transporters may need a certain NBS to always hydrolyze first in order to optimize the transport of complex substrates [17], [16]. Here we show, for the first time, that many residue positions that contribute to degeneracy in eukaryotic transporters may act as molecular switches, in order to control which NBS will hydrolyze MgATP. Therefore, the identification of these switches in our simulations of a bacterial ABC exporter may provide a novel basis to rationalize degeneracy in eukaryotic transporters. This proposal may be explored further by extended MD simulations of e.g. recent eukaryotic ABC transporter structures [50].

## Supporting Information

Figure S1 Time-resolved graphs of interactions at the MgATP binding sites. Selected atomic contacts at the MgATP binding sites. Data is plotted separately for the two binding sites in the seven simulations.
(2.04 MB TIF)

Figure S2 Time-resolved graphs of interactions at the NBD-TMD interface. Selected atomic contacts of Q-loop residues Q422-D423 plotted separately for the two NBDs in the seven simulations.
(1.35 MB TIF)

Figure S3 Interactions of the x-loop GERG motif. Scatter plots of contacts formed by residues E473-R474 of the x-loop GERG motif and the TMDs. Contacts observed in the protonated Sav1866 crystal structure are indicated by green dots. Scatter plots were generated as described for Figure 3 of the main text and SI Text S1.
(0.31 MB TIF)

Figure S4 Asymmetric stacking of H204 and R474. Coordinates along the symmetry axis of Sav1866 (z-coordinates) of selected residues in the seven repeat simulations.
(0.30 MB TIF)

Figure S5 Example rearrangement of residue pairs along the Sav1866 symmetry axis.
(1.88 MB TIF)

Figure S6 Schematic overview of studied interactions. One-nanosecond windows are represented by dots. R474 is defined to be closer to the TMDs if its z-position is different by >2 Å to the other R474 z-position. Q-loop Q422-MgATP interactions are colored by three different contact levels: <5 contacts blue, >5 and <14 contacts green, >14 contacts red. Q-loop D423 interactions to x-loop R474 and to K483 are colored by two levels: <1 contact blue, >1 contact red. Q-loop D423 interactions to TMD CL2 are colored: <8 contacts blue, >8 contacts red. All contact values are determined as the mean value over the respective 1 ns window.
(0.25 MB TIF)

Figure S7 Interactions at the ADP binding site in the ADP-bound control simulation. Figure was created in the same fashion as Figure 3 of the main text, but using the two 30 ns ADP-bound simulations.
(0.13 MB TIF)

Figure S8 Interactions at the NBD-TMD interface in the ADP-bound control simulation. Figure was created in the same fashion as Figure 4 of the main text, but using the two 30 ns ADP-bound simulations.
(0.17 MB TIF)

Figure S9 Functionally important interactions in a simulation of the KcsA K+ channel. Distance between the delta-C of protonated E71 and the gamma-C of D80, and E71-D80 contact numbers, derived from a KcsA simulation produced in our laboratory (P. W. Fowler and M. S. P. Sansom, unpublished) and plotted separately for the four monomers. The E71-D80 interaction is important for KcsA slow inactivation [50], and is presumably required to form symmetrically for proper function of the K+ selectivity filter.
(0.36 MB TIF)

Text S1 Snapshots from the simulations. Snapshots show the protein, MgATP, and the 40 closest water molecules to MgATP. The PDB file contains six snapshots as separate models. All are from one simulation, at times 0, 5, 10, 20, 30, and 40 ns.
(4.79 MB TXT)

Text S2 Additional details of method used for generation of scatter plots (for Figures 3B–E, 4A–D of main text, & SI Figure S3).
(0.11 MB DOC)
