# Supplementary figures and images for: Asymmetric Switching in a Homodimeric ABC Transporter: A Simulation Study
Source: PLoS Comput Biol. 2010 Apr 29;6(4):e1000762. doi: 10.1371/journal.pcbi.1000762 (PMC2861673; doi:10.1371/journal.pcbi.1000762)

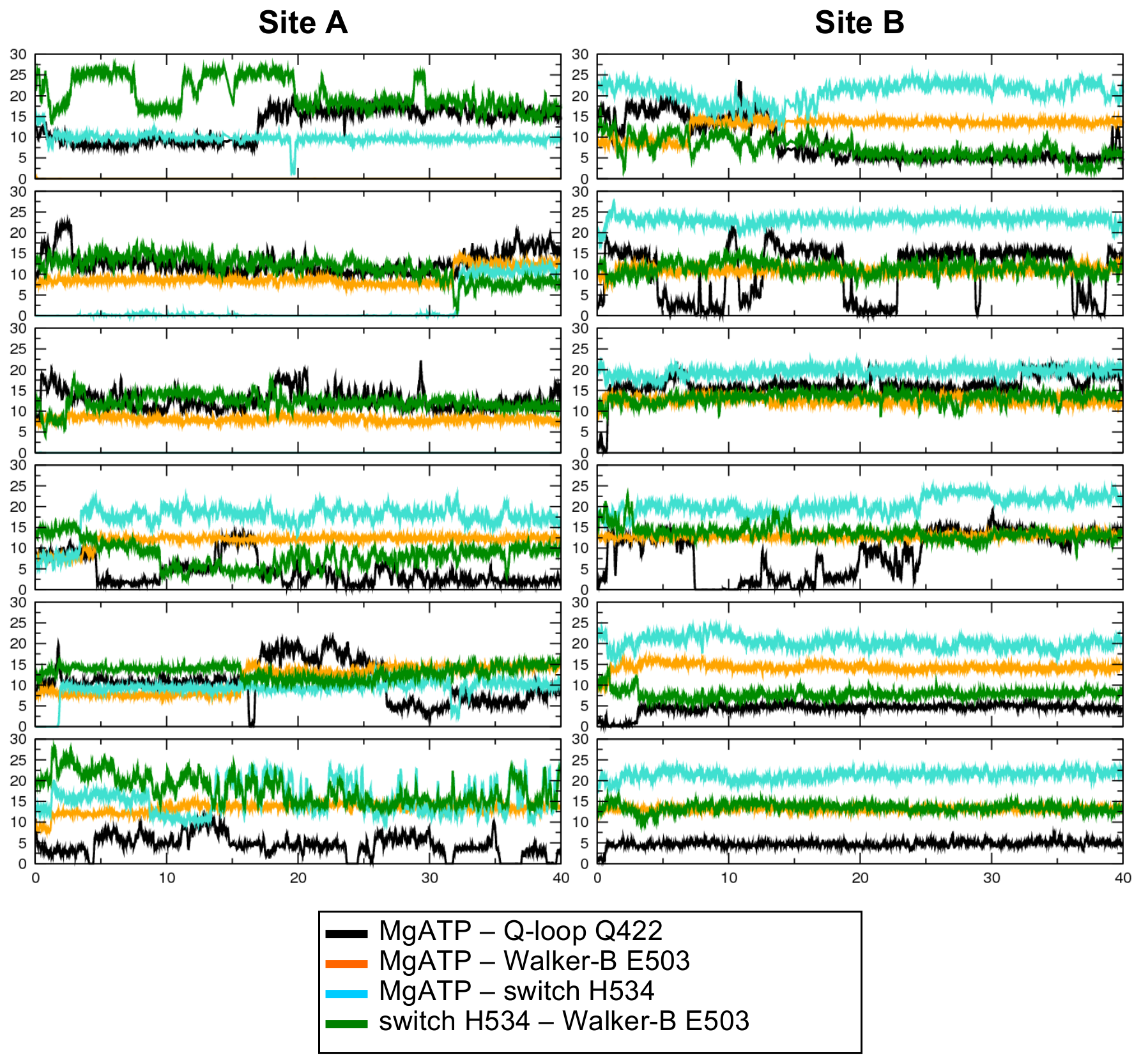

Supplement: Figure S1 — Time-resolved graphs of interactions at the MgATP binding sites. Selected atomic contacts at the MgATP binding sites. Data is plotted separately for the two binding sites in the seven simulations. (2.04 MB TIF) [file pcbi.1000762.s001.tif]

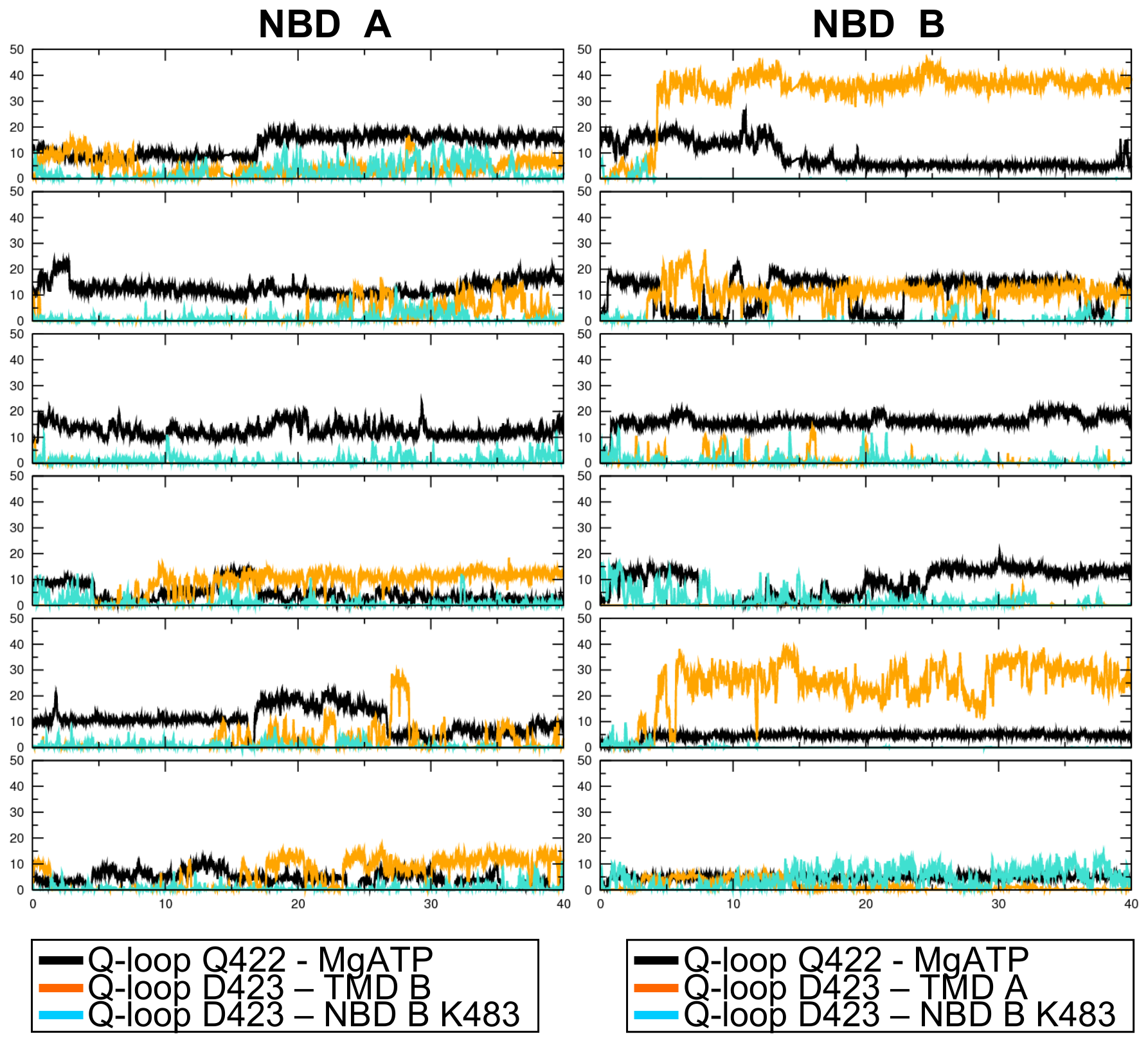

Supplement: Figure S2 — Time-resolved graphs of interactions at the NBD-TMD interface. Selected atomic contacts of Q-loop residues Q422-D423 plotted separately for the two NBDs in the seven simulations. (1.35 MB TIF) [file pcbi.1000762.s002.tif]

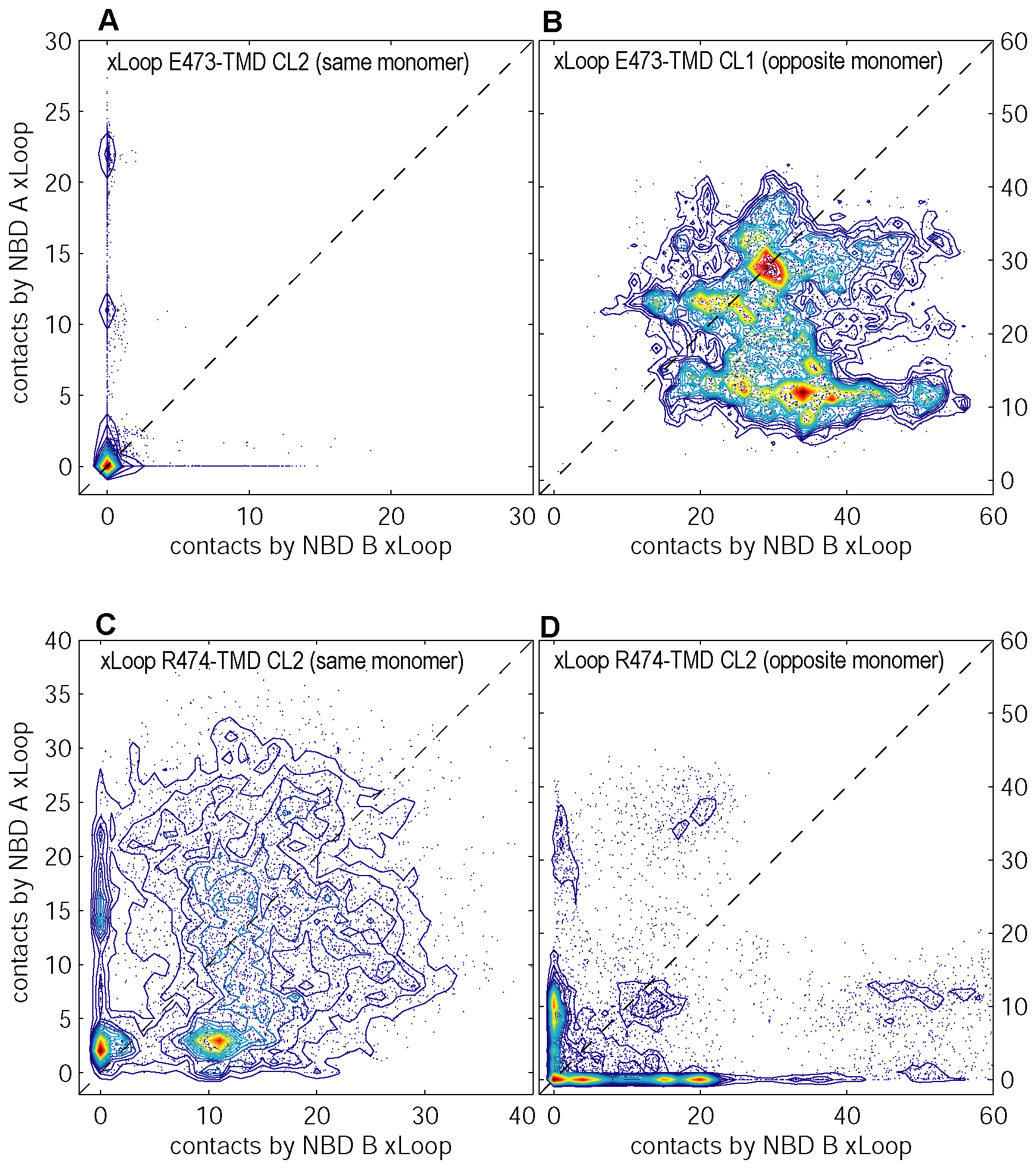

Supplement: Figure S3 — Interactions of the x-loop GERG motif. Scatter plots of contacts formed by residues E473-R474 of the x-loop GERG motif and the TMDs. Contacts observed in the protonated Sav1866 crystal structure are indicated by green dots. Scatter plots were generated as described for Figure 3 of the main text and SI Text S1. (0.31 MB TIF) [file pcbi.1000762.s003.tif]

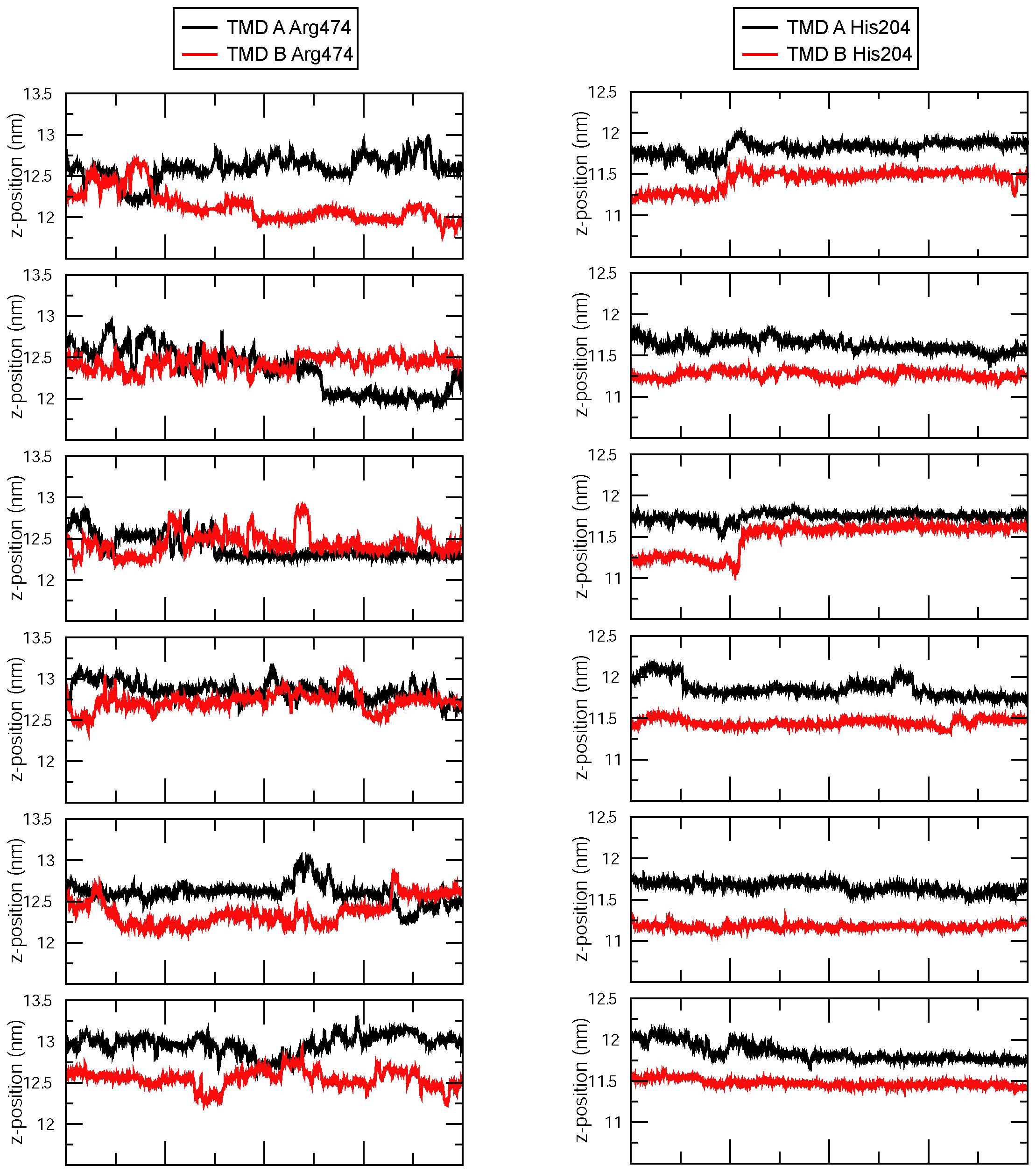

Supplement: Figure S4 — Asymmetric stacking of H204 and R474. Coordinates along the symmetry axis of Sav1866 (z-coordinates) of selected residues in the seven repeat simulations. (0.30 MB TIF) [file pcbi.1000762.s004.tif]

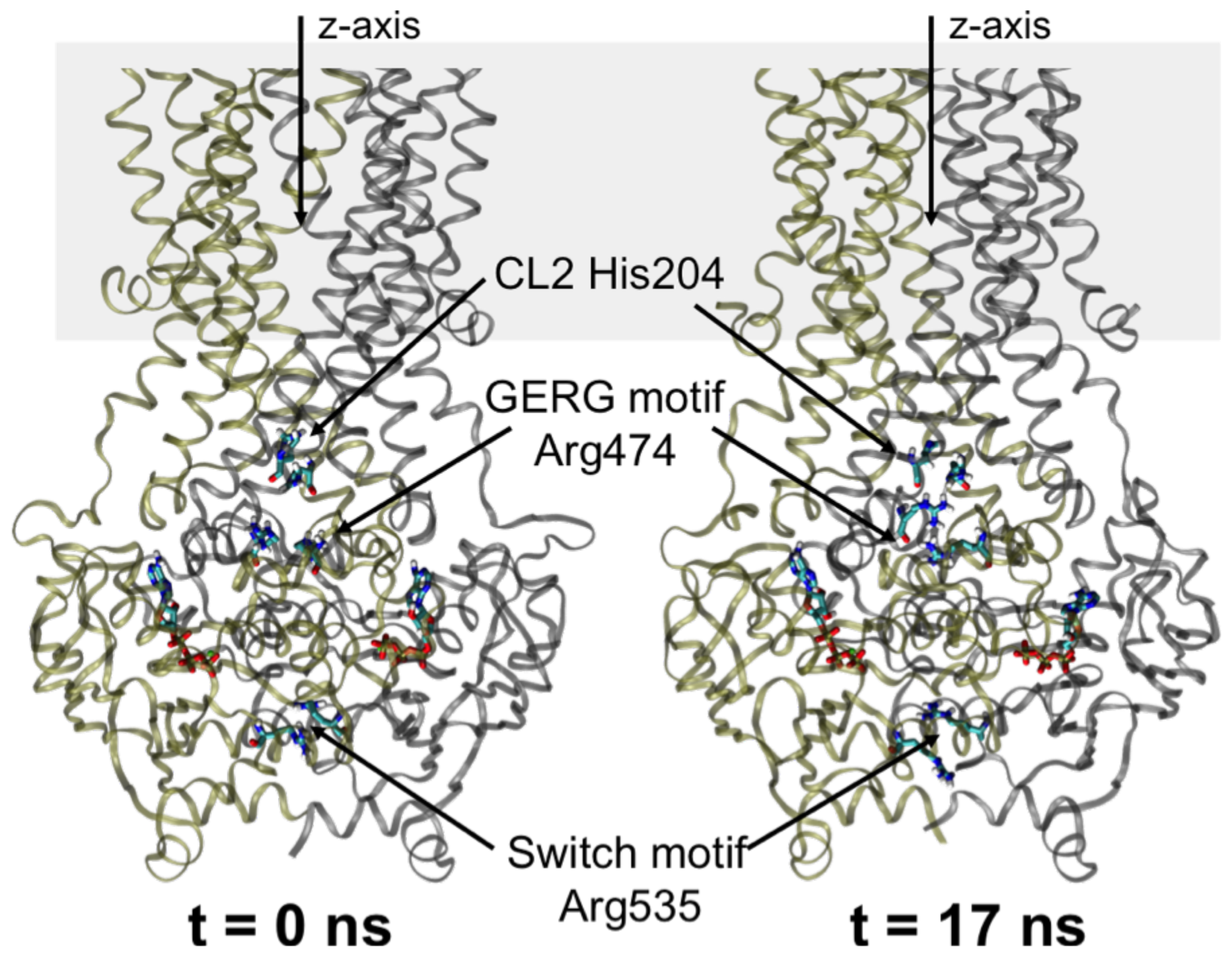

Supplement: Figure S5 — Example rearrangement of residue pairs along the Sav1866 symmetry axis. (1.88 MB TIF) [file pcbi.1000762.s005.tif]

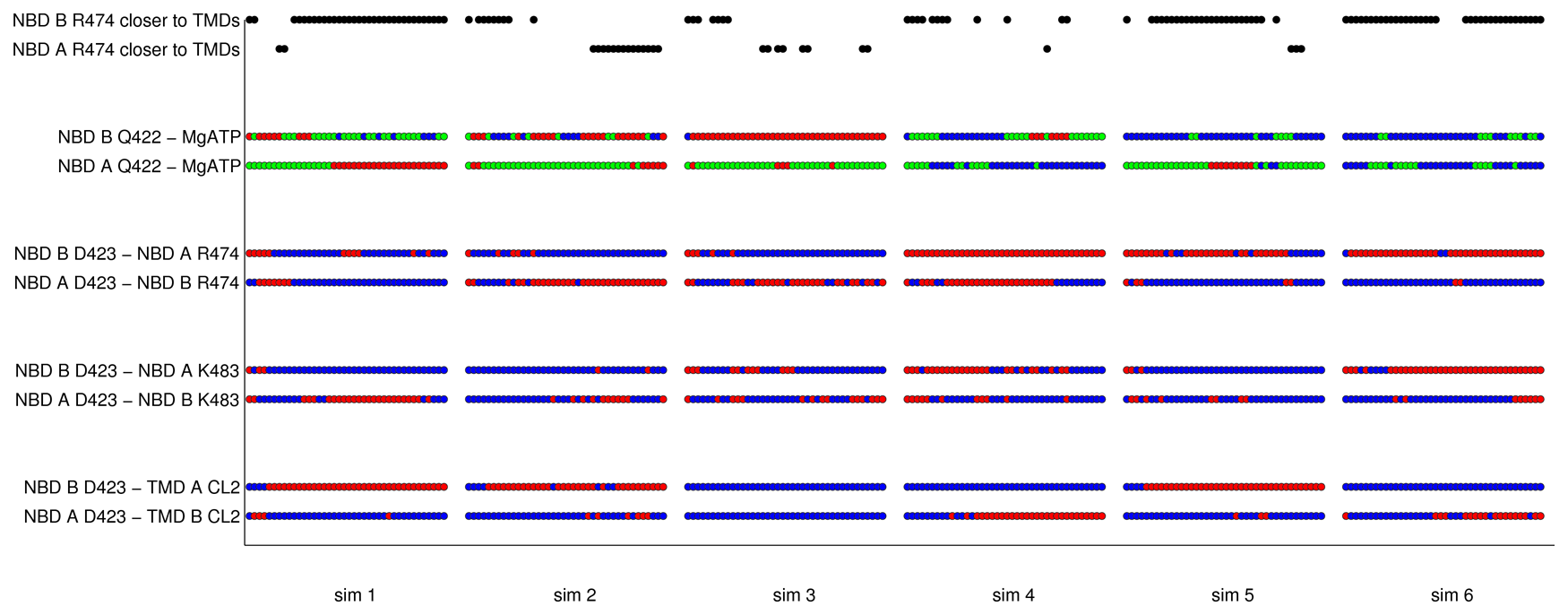

Supplement: Figure S6 — Schematic overview of studied interactions. One-nanosecond windows are represented by dots. R474 is defined to be closer to the TMDs if its z-position is different by >2 Å to the other R474 z-position. Q-loop Q422-MgATP interactions are colored by three different contact levels: <5 contacts blue, >5 and <14 contacts green, >14 contacts red. Q-loop D423 interactions to x-loop R474 and to K483 are colored by two levels: <1 contact blue, >1 contact red. Q-loop D423 interactions to TMD CL2 are colored: <8 contacts blue, >8 contacts red. All contact values are determined as the mean value over the respective 1 ns window. (0.25 MB TIF) [file pcbi.1000762.s006.tif]

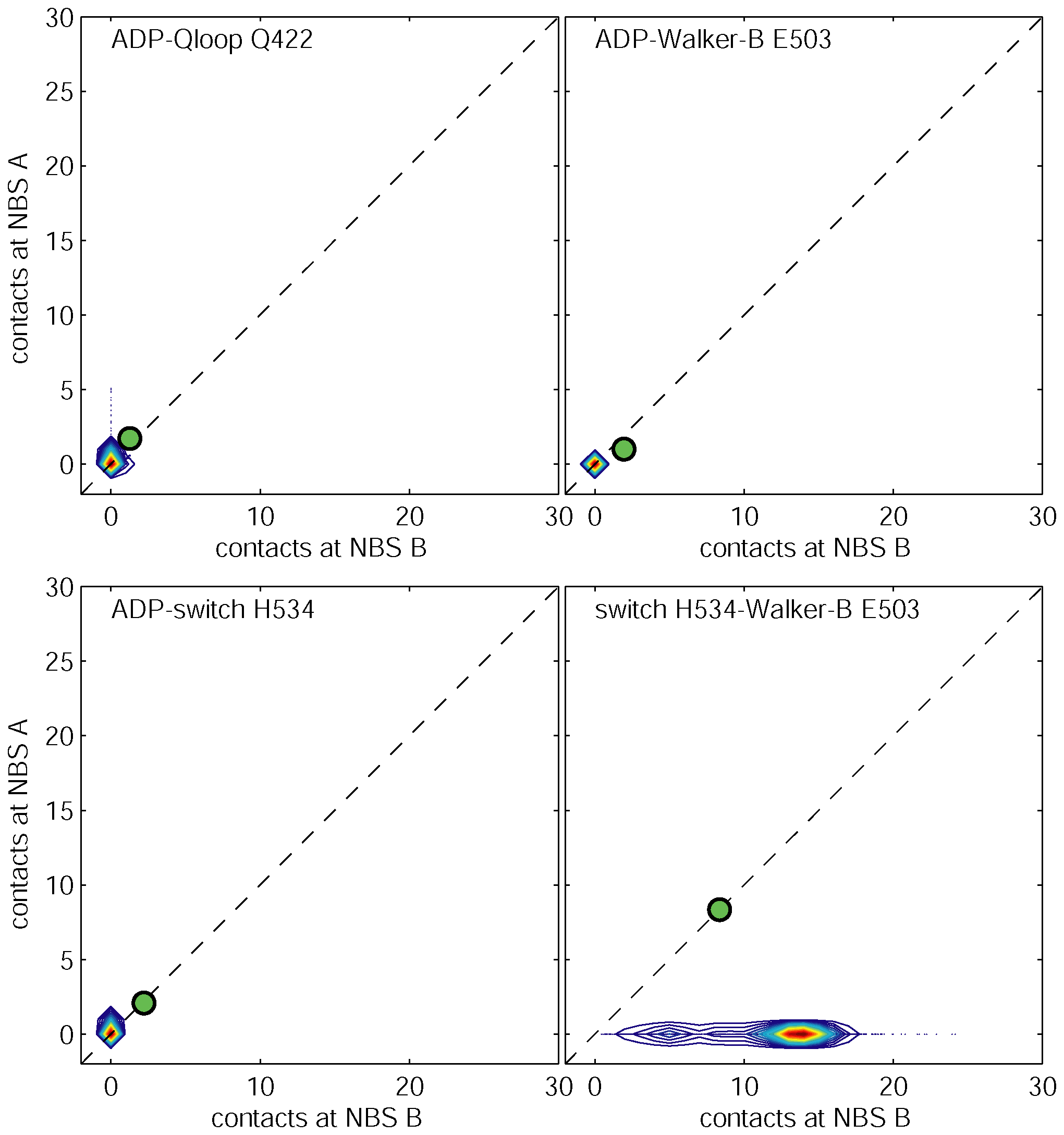

Supplement: Figure S7 — Interactions at the ADP binding site in the ADP-bound control simulation. Figure was created in the same fashion as Figure 3 of the main text, but using the two 30 ns ADP-bound simulations. (0.13 MB TIF) [file pcbi.1000762.s007.tif]

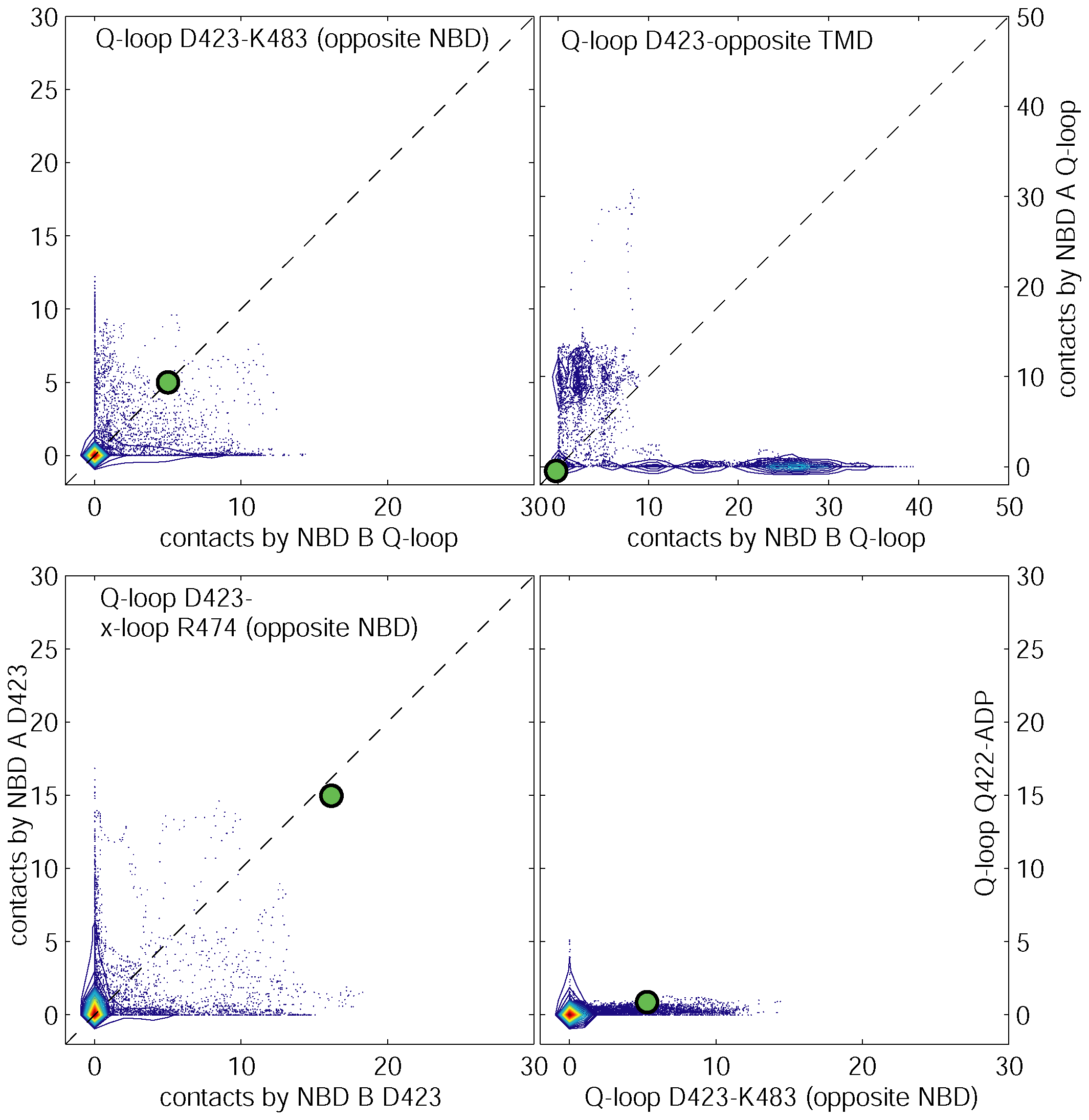

Supplement: Figure S8 — Interactions at the NBD-TMD interface in the ADP-bound control simulation. Figure was created in the same fashion as Figure 4 of the main text, but using the two 30 ns ADP-bound simulations. (0.17 MB TIF) [file pcbi.1000762.s008.tif]

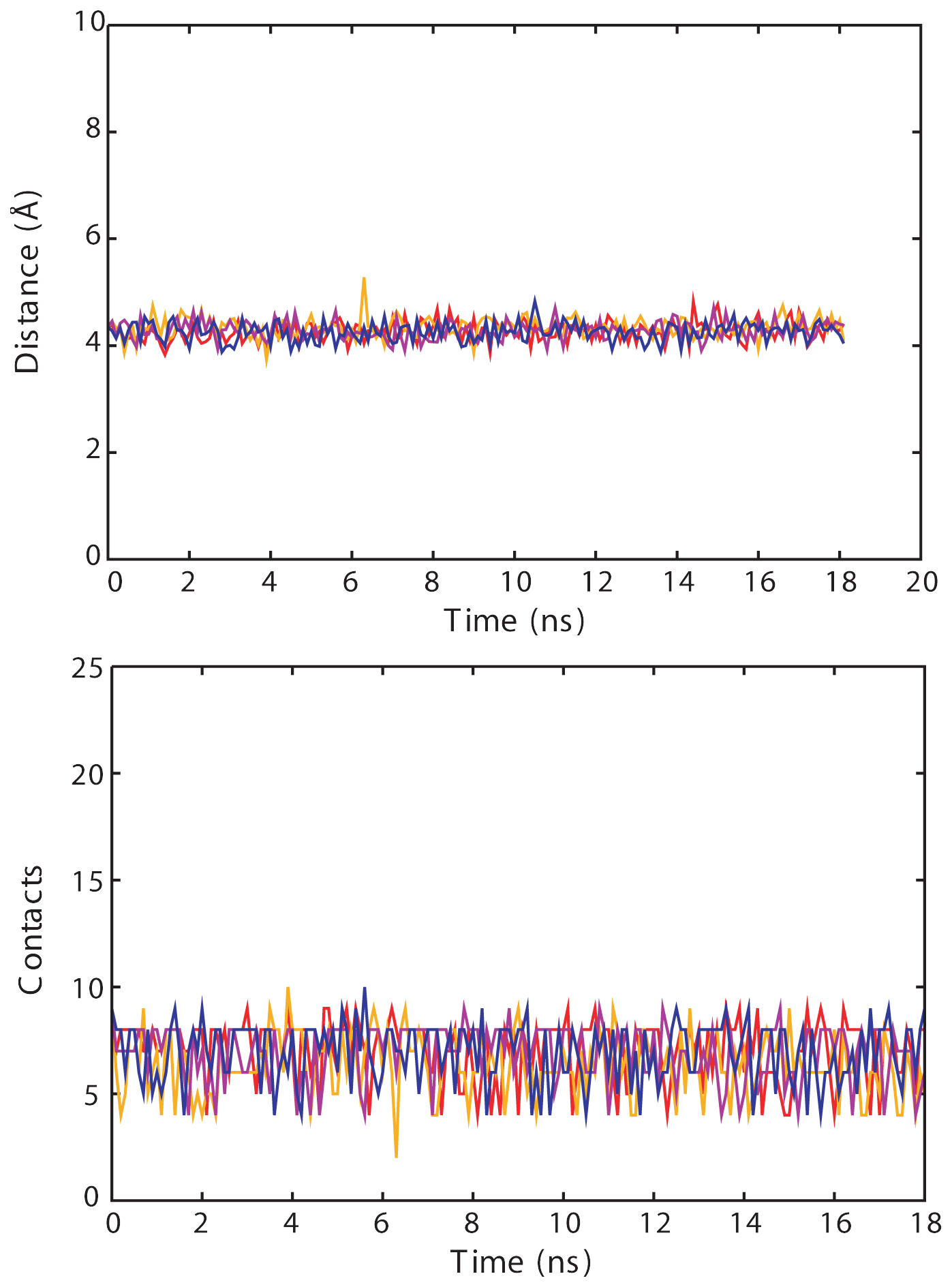

Supplement: Figure S9 — Functionally important interactions in a simulation of the KcsA K+ channel. Distance between the delta-C of protonated E71 and the gamma-C of D80, and E71-D80 contact numbers, derived from a KcsA simulation produced in our laboratory (P. W. Fowler and M. S. P. Sansom, unpublished) and plotted separately for the four monomers. The E71-D80 interaction is important for KcsA slow inactivation [50], and is presumably required to form symmetrically for proper function of the K+ selectivity filter. (0.36 MB TIF) [file pcbi.1000762.s009.tif]
