# Supplementary material for: Asymmetric Switching in a Homodimeric ABC Transporter: A Simulation Study
Source: PLoS Comput Biol. 2010 Apr 29;6(4):e1000762. doi: 10.1371/journal.pcbi.1000762 (PMC2861673; doi:10.1371/journal.pcbi.1000762)
Supplement: Text S2 — Additional details of method used for generation of scatter plots (for Figures 3B–E, 4A–D of main text, & SI Figure S3). (0.11 MB DOC) [file pcbi.1000762.s011.doc]

**Scatter plot generation (Figures 3B-E, 4A-D of main text, SI Figure S3):**

Datasets for the scatter plots were generated by calculating the respective contact numbers at both NBS for every repeat simulation. Contact numbers were calculated at 5 ps intervals, and the resulting data was subjected to a running average of window length 20. Data from all simulations was concatenated separately for each NBS, with the exception of Fig. 4D for which data from both NBS was concatenated into the same dataset.

To generate a scatter plot, the respective two datasets (ie, contact data for the two NBS except in the case of Fig. 4D) were loaded into MATLAB (The Mathworks, Inc.). A dot in the scatter plot was placed for datapoints at 50 ps intervals. Contour lines at 20 evenly spaced intervals were added by the MATLAB function contour. The colors of the contour lines are chosen from a blue (lowest density of datapoints) - green - yellow - red (highest density of datapoints) scale, such that most frequently populated states are highlighted by the reddest contour lines. Green dots were added manually at contact number positions observed in the Sav1866 crystal structure, after protonating the structure in the same fashion as for the actual simulations.
